# The impact of occupational experience on cognitive and physical functional status among older adults in a representative sample of Korean subjects

**DOI:** 10.1186/s40557-015-0057-0

**Published:** 2015-03-20

**Authors:** Jin-young Min, Jae Bum Park, Kyung-jong Lee, Kyoung-bok Min

**Affiliations:** Institute of Health and Environment, Seoul National University, Chongno-gu Yongeun-dong 28, Seoul, Republic of Korea; Department of Occupational & Environmental Medicine, Ajou University School of Medicine, San 5, Wonchon-dong, Yeongtong-gu, Suwon, 443-721 Republic of Korea; Department of Preventive Medicine, College of Medicine, Seoul National University, Seoul, Republic of Korea

## Abstract

**Objective:**

This study investigated the impact of occupation on cognitive and physical function within an occupational window of the past 15 years using a nationally representative sample in Korea.

**Methods:**

A total of 4,408 subjects aged 60 and older were selected from the Korean Longitudinal Study of Aging (KLoSA). Functional health was evaluated using the Korean versions of the Mental State Examination (K-MMSE), Instrumental Activities of Daily Living (K-IADL), and Activities of Daily Living (K-ADL) measures.

**Results:**

The prevalence of lower function was higher among women than among men, and employed persons had better cognitive and physical function compared with the retired and unemployed. Longer work duration during the past 15 years significantly and positively affected all measures of function in men, whereas it only improved physical function in women. Manual laborers exhibited improved functional capacity with longer work durations over the past 15 years, although they presented lower functional scores compared with non-manual laborers. There was a curvilinear relationship of work duration with cognitive and physical function among men and manual laborers.

**Conclusion:**

In our population, longer work duration, especially for men and for manual laborers, appears to be a significant contributor to the cognitive and physical function of older people.

## Introduction

With an aging population worldwide, there has been much interest in how to age ‘successfully.’ Although there is no clear consensus on the definition of successful aging, physical and mental health status, namely the absence of physical disability and cognitive impairment, appear to be critical for contextualizing successful aging [11].

In short, health is a positive concept emphasizing the fulfillment of integrated functions including social well-being, personal resources, and physical fitness [24]. Health status can be affected by several factors, such as personal behaviors and socioeconomic status, in a variety of ways [9,19]; among these, socioeconomic factors may be more fundamental in that they enable people to access important resources that can be used to minimize disease risk or affect health outcomes [9,24].

Interestingly, the socioeconomic factors seem to impact on health differently over the course of a lifetime [35]. For example, at early stages of life, parental education and home environment affect infants’ and children’s development and health [7,12]. In adolescence, parental concerns over education and family conflict are associated with certain health problems [34]. In adult life, it is unsurprising to find that social class, determined to a large extent by education and occupational status, and family environment are associated with health status [30]. Thus, some potential risk factors for poor health outcomes earlier in life appear to become less important later in life, while some other factors become even more influential. Which of these social factors are responsible for maintaining and promoting overall function in older populations? Occupation may be one of the main contributors to older people’s health and well-being. This is because occupation in elderly populations not only provides economic independence but also continuously provides social support and emotional recognition, even though there are also certain mediating factors, such as psychosocial stress and exposure in the workplace [23,24,32]. Therefore, the benefits (i.e., economic independence, social support, and emotional recognition) may translate directly or indirectly into better health later in life.

However, since good health contributes to the chances of securing or remaining in a paid job–the ‘healthy worker’ hypothesis–the correlation may represent an effect of individual health, rather than an effect of occupation [26]. To more adequately address the influence of occupation on health, it is necessary to trace the occupational record, rather than simply looking at a single time point. Many older persons want or need to work even after voluntary or mandatory retirement, for economic and psychological reasons; the burning question is whether this is better for their health and well-being, and more information is needed to understand this issue.

Despite the growing importance of occupation in the geriatric population in Korea, there is little evidence on the specific relationship between occupation and the health of older persons. In light of this, we investigated the impact of occupation on cognitive and physical function within an occupational window covering the past 15 years, using a nationally representative sample in Korea. We hypothesized that employment would have different effects on cognitive and physical function depending on sex and job types.

## Materials and methods

### Study population

This study used a sample from the first-wave data of the Korean Longitudinal Study of Aging (KLoSA). The KLoSA is being conducted by the Korea Labor Institute to collect the basic data needed to devise and implement effective social and economic policies to address emerging trends related to population aging. It will be repeated every even-numbered year. This original KLoSA study population comprised South Korean adults aged 45 or older and living in 15 large administrative areas. In the first baseline survey in 2006, 10,254 individuals in 6,171 households (1.7 per household) were interviewed using the Computer-Assisted Personal Interviewing method (http://www.kli.re.kr/klosa/ko/main/main.jsp) [36].

Our study sample focused on the elderly, and we excluded individuals younger than 60 (n = 4,709) or with missing questionnaire responses (n = 1,137); the final sample consisted of 4,408 older subjects (1,922 men and 2,486 women).

### Measurements

As outcome variables, the Korean versions of the Mental State Examination (K-MMSE) for cognitive function, and Instrumental Activities of Daily Living (K-IADL) and Activities of Daily Living (K-ADL) measures for physical function were used [20,37]. The K-MMSE includes 11 items divided into two sections; it incorporates both verbal and written responses to questions measuring orientation in time and space, memory, and attention. We adopted the standard cut-off of 17 or less for poor function. The K-ADL was developed to assess basic activities of the elderly, including dressing, washing (face/teeth/hair), bathing/showering, eating, getting out of bed/room, using the toilet, and controlling urination. The K-IADL was used to estimate more complex activities necessary for independent daily life. These include personal grooming, household chores, preparing meals, doing laundry, going out within a short distance, using transportation, shopping, managing money, making phone calls, and taking medication. The ADL was used to measure disability, which was defined as needing help with at least one activity; the cutoff for disability was an IADL score below 7.

Key independent variables were current employment status (‘employed’, currently have a job; ‘retired’, retired and currently have no job; and ‘unemployed’, never worked in the past 15 years), work duration during the past 15 years, job type, sociodemographic variables, and general health information. Respondents recorded their occupational histories, including work duration and job type, during their lifetime. From these data, job types were assigned based on principal lifetime occupation and classified as non-manual work (i.e., managers and professions), manual work (i.e., agriculture and machine operators), and other work (i.e., service and soldier). Work duration during the past 15 years was classified into four categories; unemployment (never worked in the past 15 years), less than 7 years, 8 to 15 years, and 15 years (worked continuously during the past 15 years). Sociodemographic variables included age, marital status, educational level, and household income. General health information included smoking and drinking habits, chronic illness, and body mass index (BMI).

### Statistical analysis

Because of the differences in occupation and functional capacity between the sexes, data are presented descriptively for each sex with means or proportions. Logistic regression models were used to examine associations between each of the three functional status measures and work duration in the past 15 years. Incremental models were formulated for each outcome, beginning with age and sex, and adding education, marital status, household income, and finally, all of the covariates including chronic diseases, smoking, and drinking. Odds ratios and 95 percent confidence intervals were generated for each model. In addition, a general additive model (GAM) was used for further analysis. The GAM is a semi-parametric extension of the general linear model and replaces the linear form ∑ ß_*j*_ X_*j*_ by a sum of smoth functions ∑ s _*j*_ (X _*j*_) [18]. The s _*j*_ (·)’s are unspecified functions that are estimated using a scatterplot smoother, in an iterative procedure. All statistical analysis was performed using the SAS 9.2 (SAS Institute, Cary, NC, USA) and R (R Foundation for Statistical Computing, Vienna, Austria).

## Results

Table 1 presents the distribution of variables of interest together with cognitive and physical function of the study population (aged 60 years or more), stratified by sex. Mean age (±SD) was 70 (±7) years (range 60–105 years) in the overall population; the mean age was the same for both sexes. The vast majority of men (91.2%) were still married, whereas less than 54.5% of women were still married and 43.7% were widowed. Education level varied largely by sex; more women than men had a low educational level, with over 43% of women never receiving any formal education, and approximately 6.5% having completed at least 12 years of schooling. Most men (86.1%) had some formal education, and 36.2% of them had completed high school or more. Over 25.8% of men and 34% of women reported an annual household income > 3 million won (1st quartile), while 15.3% of men and 13.2% of women reported an income of more than 30 million won (4th quartile). Overall, 35.9% of men and 13.5% of women were currently employed, and a large proportion (68.3%) of the women was unemployed. Regarding the subjects’ principal work categories, 5.4% of women were non-manual workers whereas approximately 24.3% of men were non-manual workers. The proportion of manual laborers among men and women was 63.1% and 66.5%, respectively. Most women (95.5%) were nonsmokers, but 58.9% of men were current or past smokers. More than half of the men (55.1%) consumed alcohol, whereas only 11.1% of women were drinkers. The proportion of subjects with hypertension was higher than for other chronic diseases and reached 32.6% for men and 39.9% for women. The proportion of men with cerebral vascular disease was about two-fold higher than for women. The proportion of overweight or obesity was similar in both men (46.4%) and women (47%); less than 7% of both male and female subjects were underweight. A greater proportion of women than of men reported low cognitive performance and limited physical function; the proportion of low scores among women was 23.9% for the MMSE, 4.1% for the IADL, and 7.2% for the ADL.

**Table 1:** **Distribution of variables of interest and cognitive and physical function by sex**

**Variables**		**Male**		**Female**	
		**n**	**(%)**	**n**	**(%)**
Marital status					
	Married	1752	(91.2)	1354	(54.5)
	Divorced	32	(1.7)	38	(1.5)
	Widowed	133	(6.9)	1086	(43.7)
	Others	5	(0.3)	7	(0.3)
Educational level					
	No education	267	(13.9)	1070	(43.0)
	Elementary	618	(32.2)	953	(38.3)
	Middle school	341	(17.7)	262	(10.5)
	High school	446	(23.2)	161	(6.5)
	College or more	249	(13.0)	40	(1.6)
Household income*					
	1st quartile	496	(25.8)	844	(34.0)
	2nd quartile	636	(33.1)	781	(31.4)
	3rd quartile	495	(25.8)	532	(21.4)
	4th quartile	295	(15.3)	329	(13.2)
Current employment status					
	Employed	690	(35.9)	336	(13.5)
	Retired	785	(40.8)	453	(18.2)
	Unemployed	447	(23.3)	1697	(68.3)
Principal job type during lifetime					
	Non-manual work	468	(24.3)	135	(5.4)
	Manual work	1212	(63.1)	1653	(66.5)
	Other	246	(12.8)	699	(28.1)
Smoking					
	Current smoker	638	(33.2)	91	(3.7)
	Past smoker	494	(25.7)	20	(0.8)
	Nonsmoker	790	(41.1)	2375	(95.5)
Drinking					
	Yes	1059	(55.1)	277	(11.1)
	No	863	(44.9)	2209	(88.9)
Chronic disease					
	Hypertension	627	(32.6)	993	(39.9)
	Diabetes mellitus	295	(15.3)	400	(16.1)
	Cardiac disorder	122	(6.3)	187	(7.5)
	Cerebral vascular disease	115	(6.0)	87	(3.5)
BMI¶					
	Severe obesity	22	(1.1)	67	(2.7)
	Obesity	321	(16.7)	499	(20.1)
	Overweight	550	(28.6)	602	(24.2)
	Normal	881	(45.8)	1040	(41.8)
	Underweight	111	(5.8)	150	(6.0)
MMSE					
	≤17	176	(9.2)	593	(23.9)
	>17	1746	(90.8)	1893	(76.1)
	*Mean (SD)*	24.33	(5.80)	21.12	(6.83)
IADL					
	≤7	1846	(96.0)	2385	(95.9)
	>7	76	(4.0)	101	(4.1)
	*Mean (SD)*	0.78	(2.11)	0.81	(2.14)
ADL					
	<1	1804	(93.9)	2307	(92.8)
	≥1	118	(6.1)	179	(7.2)
	*Mean (SD)*	0.26	(1.18)	0.23	(1.01)

*Household income: 1st quartile, below 3 million won; 2nd quartile, between 3 million and 12 million won; 3rd quartile, between 12 million and 30 million won; 4th quartile, more than 30 million won.

¶BMI(body mass index): severe obesity, BMI ≥30; obesity, 30 < BMI ≤ 25; overweight, 25 < BMI ≤ 23; normal,23 < BMI ≤ 18.5; underweight, BMI < 18.5.

Tables 2 and 3 show the results of logistic analysis of work duration and functional status according to sex and job categories. For men, increased work duration was associated with higher performance on the MMSE (*P* for trend = 0.0151), IADL (*P* for trend = 0.0007), and ADL (*P* for trend = 0.0002), whereas extended work duration for women only improved physical function scores (IADL *P* for trend = 0.0449 and ADL *P* for trend = 0.0362). Among the three job categories, manual workers showed a positive, significant relationship between work duration and all three measures of function in *P*-values for trend analysis (MMSE *P* for trend = 0.0328; IADL *P* for trend = 0.0044; ADL *P* for trend = 0.0004).

**Table 2:** **Logistic analysis of work duration and functional status by sex**

**Work duration (year)**	**Men**			**Women**			
	**Model 1**	**Model 2**	**Model 3**	**Model 1**	**Model 2**	**Model 3**	
MMSE							
	15	0.532	0.320	0.429	1.598	0.988	0.934
		(0.326,0.869)	(0.190,0.538)	(0.243,0.759)	(1.205,2.118)	(0.730,1.336)	(0.677,1.288)
	8-14	0.658	0.469	0.533	1.512	1.048	1.037
		(0.400,1.083)	(0.277,0.793)	(0.300,0.946)	(1.122,2.038)	(0.760,1.445)	(0.739,1.455)
	1-7	0.507	0.452	0.425	1.165	0.904	0.854
		(0.299,0.862)	(0.259,0.790)	(0.228,0.790)	(0.868,1.565)	(0.660,1.237)	(0.611,1.192)
	0	Reference	Reference	Reference	Reference	Reference	Reference
*P* for trend	0.038	0.0003	**0.0151**	0.0037	0.8781	0.7441	
IADL							
	15	0.135	0.100	0.140	0.236	0.187	0.230
		(0.057,0.323)	(0.041,0.243)	(0.051,0.383)	(0.083,0.667)	(0.065,0.533)	(0.079,0.665)
	8-14	0.702	0.584	0.643	1.053	0.900	0.935
		(0.371,1.327)	(0.306,1.115)	(0.301,1.370)	(0.558,1.989)	(0.472,1.719)	(0.462,1.894)
	1-7	0.463	0.449	0.382	1.237	1.157	1.117
		(0.231,0.931)	(0.221,0.911)	(0.164,0.892)	(0.712,2.146)	(0.661,2.023)	(0.604,2.067)
	0	Reference	Reference	Reference	Reference	Reference	Reference
*P* for trend	<.0001	<.0001	**0.0007**	0.0308	0.0121	**0.0449**	
ADL							
	15	0.163	0.125	0.196	0.432	0.352	0.414
		(0.085,0.313)	(0.065,0.243)	(0.095,0.404)	(0.244,0.767)	(0.197,0.631)	(0.223,0.767)
	8-14	0.526	0.459	0.495	0.992	0.879	0.974
		(0.304,0.910)	(0.263,0.800)	(0.262,0.935)	(0.622,1.581)	(0.547,1.413)	(0.585,1.621)
	1-7	0.569	0.545	0.494	1.032	0.953	1.011
		(0.328,0.987)	(0.312,0.954)	(0.257,0.947)	(0.672,1.587)	(0.616,1.473)	(0.628,1.628)
	0	Reference	Reference	Reference	Reference	Reference	Reference
*P* for trend	<.0001	<.0001	**0.0002**	0.029	0.0054	**0.0362**	

The reported values indicate odds ratios (95% CI).

Model 1: controlling for age.

Model 2: controlling for marital status, educational level, household income, and a component in Model 1.

Model 3: controlling for chronic disease, BMI, smoking, drinking, and components in Models 1 and 2.

Bold numbers indicate p < 0.05 in final models.

**Table 3:** **Logistic analysis of work duration and functional status by job category**

**Work duration (year)**		**Non-manual worker**			**Manual worker**			**Other workers**		
		**Model 1**	**Model 2**	**Model 3**	**Model 1**	**Model 2**	**Model 3**	**Model 1**	**Model 2**	**Model 3**
MMSE										
	15	1.168	1.098	1.174	1.107	0.825	0.803	1.136	0.869	0.950
		(0.569,2.399)	(0.519,2.323)	(0.536,2.572)	(0.895,1.369)	(0.661,1.029)	(0.637,1.012)	(0.724,1.784)	(0.541,1.395)	(0.586,1.542)
	8-14	0.670	0.692	0.766	1.440	1.127	1.106	1.238	1.030	1.074
		(0.339,1.324)	(0.335,1.432)	(0.356,1.647)	(1.141,1.818)	(0.885,1.434)	(0.861,1.420)	(0.838,1.830)	(0.680,1.559)	(0.702,1.641)
	1-7	0.861	0.737	0.756	1.062	0.948	0.917	1.051	0.843	0.784
		(0.444,1.670)	(0.365,1.491)	(0.356,1.603)	(0.839,1.345)	(0.744,1.209)	(0.712,1.180)	(0.711,1.554)	(0.560,1.270)	(0.512,1.199)
	0	Reference	Reference	Reference	Reference	Reference	Reference	Reference	Reference	Reference
*P* for trend		0.3908	0.4833	0.5995	0.0119	0.0328	**0.0328**	0.7545	0.7777	0.6219
IADL										
	15	0.214	0.263	0.354	0.193	0.165	0.234	0.166	0.152	0.118
		(0.035,1.309)	(0.040,1.718)	(0.040,3.159)	(0.090,0.413)	(0.076,0.355)	(0.101,0.541)	(0.020,1.357)	(0.018,1.255)	(0.013,1.116)
	8-14	0.527	0.689	1.092	1.036	0.917	0.997	0.849	0.816	0.662
		(0.141,1.968)	(0.178,2.664)	(0.189,6.304)	(0.613,1.751)	(0.538,1.563)	(0.547,1.816)	(0.277,2.607)	(0.257,2.598)	(0.175,2.511)
	1-7	0.277	0.358	0.212	0.802	0.790	0.841	1.412	1.332	1.149
		(0.066,1.162)	(0.082,1.553)	(0.027,1.685)	(0.469,1.373)	(0.460,1.357)	(0.450,1.572)	(0.581,3.436)	(0.538,3.296)	(0.421,3.134)
	0	Reference	Reference	Reference	Reference	Reference	Reference	Reference	Reference	Reference
*P* for trend		0.2154	0.3646	0.2798	0.0001	<.0001	**0.0044**	0.2541	0.2565	0.2595
ADL										
	15	0.237	0.268	0.325	0.270	0.240	0.332	0.205	0.197	0.179
		(0.054,1.033)	(0.060,1.202)	(0.062,1.710)	(0.166,0.439)	(0.147,0.393)	(0.197,0.561)	(0.044,0.945)	(0.042,0.915)	(0.034,0.941)
	8-14	0.437	0.491	0.517	0.800	0.734	0.803	0.959	0.964	1.001
		(0.141,1.350)	(0.158,1.524)	(0.131,2.032)	(0.529,1.211)	(0.482,1.118)	(0.506,1.274)	(0.399,2.303)	(0.391,2.380)	(0.350,2.863)
	1-7	0.405	0.456	0.331	0.857	0.830	0.876	0.977	0.923	0.875
		(0.136,1.208)	(0.150,1.389)	(0.082,1.331)	(0.574,1.280)	(0.554,1.244)	(0.558,1.377)	(0.437,2.184)	(0.407,2.091)	(0.348,2.198)
	0	Reference	Reference	Reference	Reference	Reference	Reference	Reference	Reference	Reference
*P* for trend		0.1987	0.3049	0.3759	<.0001	<.0001	**0.0004**	0.2261	0.2132	0.2269

The reported values indicate odds ratios (95% CI).

Model 1: controlling for age and sex.

Model 2: controlling for marital status, educational level, household income, and a component in Model 1.

Model 3: controlling for chronic disease, BMI, smoking, drinking, and components in Models 1 and 2.

Bold numbers indicate p < 0.05 in final models.

**Figure 1:**
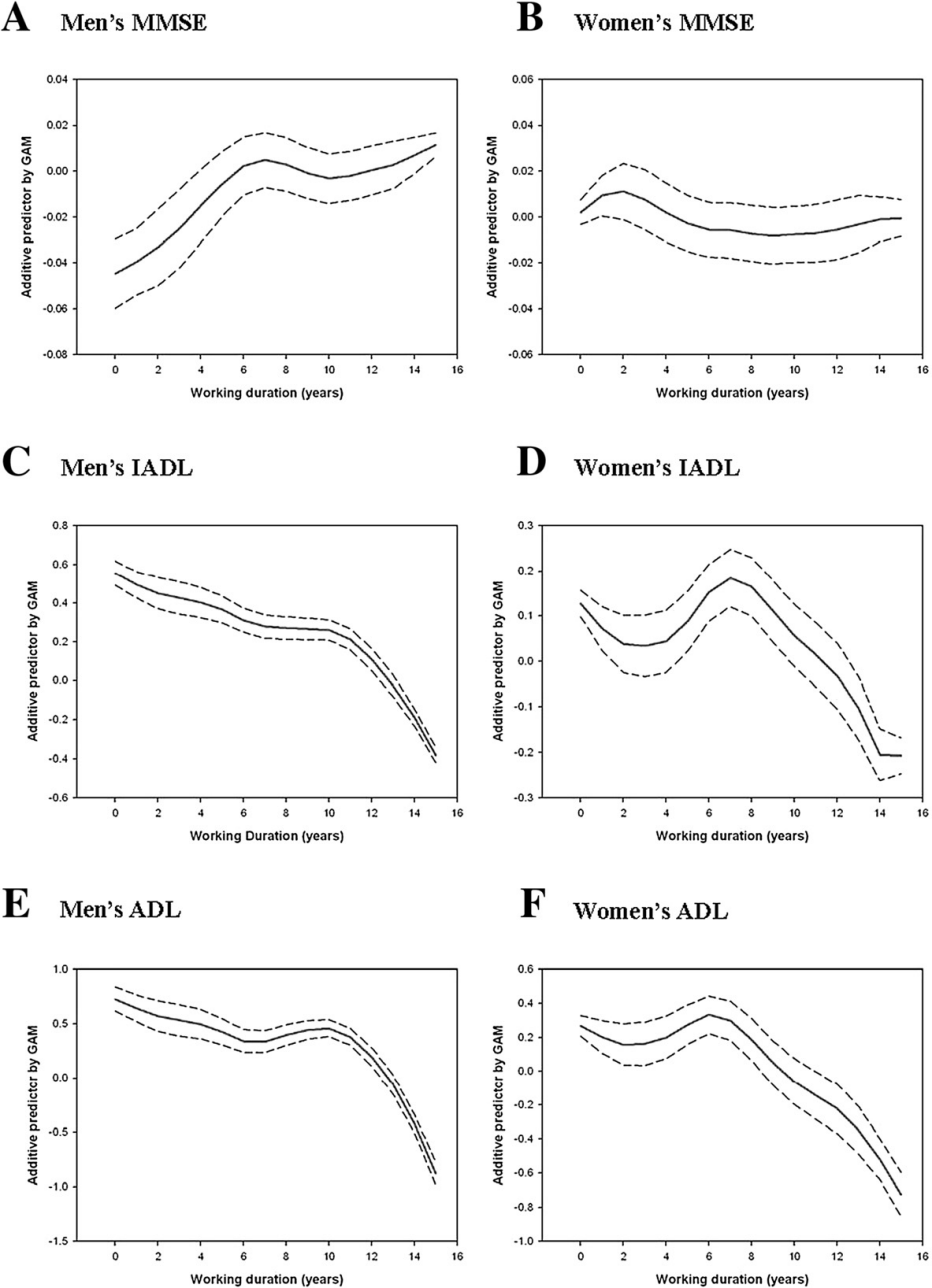
**GAM output of changes in cognitive and physical performances during the past 15 years according to sex. (A)** Men’s MMSE; **(B)** Women’s MMSE; **(C)** Men’s IADL; **(D)** Women’s IADL; **(E)** Men’s ADL; **(F)** Women’s ADL. The y-axis represents the additive predictor by GAM, which is a smoothed function for each variable and explains the effect of each variable on work duration. The rug-plot on the x-axis represents the number of observations, and dashed lines are the SE +/− 2 confidence bands.

**Figure 2:**
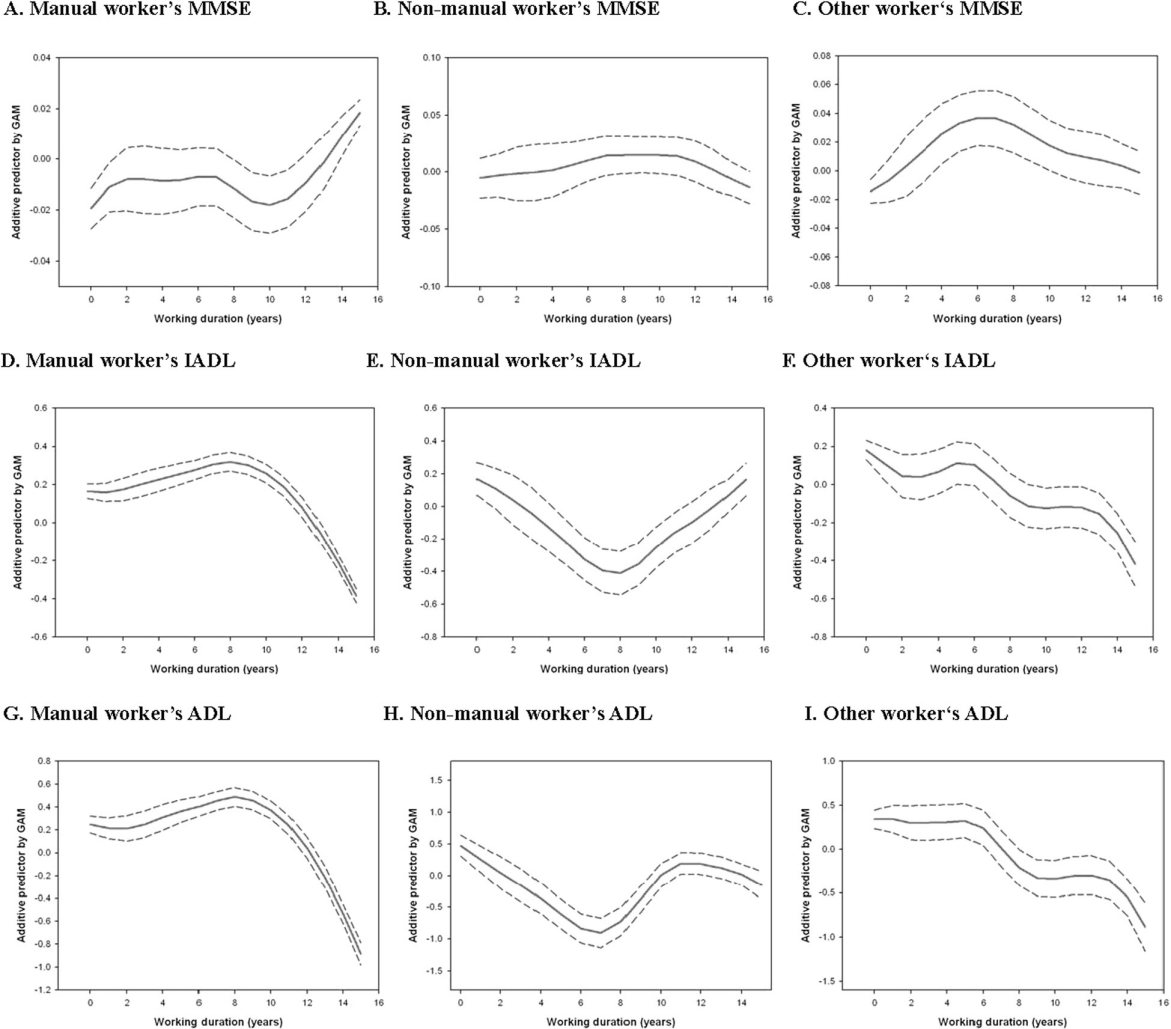
**GAM output of changes in cognitive and physical performances during the past 15 years according to job categories. (A)** Manual worker’s MMSE; **(B)** Non-manual worker’s MMSE; **(C)** Other worker‘s MMSE; **(D)** Manual worker’s IADL; **(E)** Non-manual worker’s IADL; **(F)** Other worker‘s IADL; **(G)** Manual worker’s ADL; **(H)** Non-manual worker’s ADL; **(I)** Other worker‘s ADL. The y-axis represents the additive predictor by GAM, which is a smoothed function for each variable and explains the effect of each variable on working duration. The rug-plot on the x-axis represents the number of observations, and dashed lines are the SE +/−2 confidence bands.

Figures 1 and 2 show the predicted distribution of cognitive and physical function, based on work duration over the past 15 years, according to sex and job categories. We found a curvilinear relationship between work duration and the three measures of function, namely MMSE, IADL, and ADL, among men and manual workers; the probability of better cognitive and physical performance among men and manual workers increased with work duration of more than 10 years within the past 15 years.

## Discussion

This study focused on the effect of occupation on the cognitive and physical function of older Koreans. Two main results were found. First, the influence of occupation on health differed to some extent by gender and job category; longer work duration in men contributed to better cognitive and physical function, whereas occupation in women affected only physical capacities. Participants who engaged principally in manual work had a positive effect on functional capacities with increasing work duration, although they had lower functional scores than non-manual and other workers. Second, there was a curvilinear relationship between work duration and function in men and in manual workers.

The findings are in broad agreement with those of previous studies; convincing evidence has been presented for the association between occupation and functional health among older persons. For cognitive function, Dartigues et al. [10] and Frisoni et al. [16] showed that principal occupation during one’s lifetime was strongly associated with MMSE scores as a measure of cognitive function in elderly French subjects, and that subjects who had engaged principally in blue-collar work during their lifetime had lower average scores than white-collar workers. Some researchers observed significant association between cognitive performance in elderly people and specific job characteristics, such as unskilled blue-collar work, occupational complexity, and patterns of occupational demands [1,6,27]. For physical function, the results of a longitudinal study suggested that socioeconomic disadvantage, caused by occupation and other factors, was closely linked to the initial level, onset, and progression of disability [17]. Li et al. [28] and Russo et al. [33] found a potential effect on physical function of the longest-held occupation in a lifetime in an elderly population. Furthermore, Ebrahim et al. [13] and Ramsay et al. [31] reported a strong association between disability and socioeconomic status as measured by occupational class. Many studies have tried to help clarify which physical functions are more susceptible to the occupational effect when comparing blue-collar and white-collar workers [8,22]).

Our findings are interesting in that they indicate that work experience in the past 15 years may have a more positive effect on men’s cognitive function than on women’s. Another interesting finding was that work experience in the past 15 years may help older persons engaged principally in manual work to develop better cognitive and physical function, although these individuals have poorer capacities than non-manual or other workers in general. These gaps in functional capacity based on sex and occupation (men vs. women, manual vs. non-manual or other workers) may arise from differences in social networks, conditions, or attitudes related to occupation. Studies of cognitive decline have suggested that social relations may be an influential factor. Berkman et al. [3] presented a cascading causal progression from macro-social condition, which embeds social networking in larger social and cultural structures, to psychobiological mechanisms by which social interaction affects health. Moving downstream, social networking has an effect on social and interpersonal behaviors. Thus, positive social networking may help to maintain or improve cognitive function [3,39]. However, it has been also shown that social networking through relationships with others greatly varies between men and women [2]. Women’s relationships with others have been characterized as “face to face,” with friendships based on mutuality and intimacy, while men’ relationships have been characterized as “side by side” and seem more defined by public and social boundaries [2,38]. Based on this evidence, occupation for men may be a very effective means to not only perform social networking but also to positively impact cognitive function.

Several studies have shown that manual (or blue-collar) workers face low wages, poor work conditions, physically strenuous work, and a lack of social support [15,21], all of which may erode health through direct or indirect pathways, and thus make these workers more vulnerable to poorer cognitive and physical performance than non-manual and other workers. Paradoxically, despite these disadvantages, subjects who engaged mostly in manual work during their lifetime experienced a positive health effect with increasing work duration over the past 15 years, whereas the effect among non-manual and other workers was negligible. Although the causality was not clear, this result may suggest that economic or non-economic rewards from occupation are more important for manual workers, regardless of the physical and mental demands of the workplace. For example, since manual workers are most economically affected, their payment may be a more important factor in maintaining household income and averting economic hardship than for non-manual or other workers. Also, since occupation has a feedback effect on lifestyle [14], systematic health programs in the workplace may promote the maintenance of health and function.

Substantial effort has been devoted to clarifying the impact of socioeconomic factors on health indicators in later life [4,5,25,29]). Most of all, education has been focused as a main determinant of health, due to positive and long-lasting effects on subsequent functional changes in later life [5,9,25]). Despite the importance of education [5,9,25]), however, considering the stigma associated with older people, who are among the most economically disadvantaged segments of society and are often isolated and ignored, occupation may be a better discriminator of health risk than education for older persons. Actually, there is some negative publicity related to exposure to harmful materials or stressful work conditions, but occupation seems to give rise health advantages for them [23,24]. As confirmed by our findings, the benefits of engagement in occupation in later life extend beyond the effects of labor itself, as occupation may lead to economic independence, improved self-esteem, and social integration and recognition. Although there are differences based on sex and job type, these benefits may directly or indirectly sustain better function and greater comfort.

## Conclusions

In conclusion, older Korean people in our study who continued to work exhibited improved cognitive and physical function. Our study exhibits certain limitations because cognitive function and physical function were only measured using respondents’ self-reports, without a medical examination. The tools used in this study have been widely used to estimate function in older people, but they may not reflect actual capabilities and may not adequately address functional health problems. Moreover, we assessed principal lifetime occupation and work duration during the past 15 years retrospectively, based on subjects’ occupational history record. This does not allow us to rule out the possibility of recall bias and the health worker effect. Continued follow-up of the KLoSA cohort might allow us to determine more precisely whether occupational experience is a correlate of cognitive performance and a risk factor for health among the elderly.
